# A systematic review of gastrointestinal parasites in *Sus scrofa domesticus* from Europe (2010 – 2026)

**DOI:** 10.3389/fpara.2026.1950643

**Published:** 2026-10-01

**Authors:** Alesia Cioacă, Diana Hoffman, Ioan Cristian Dreghiciu, Tiana Florea, Mirela Imre, Anamaria Plesko, Mălina Oprescu, Ion Oprescu, Gheorghe Dărăbuş, Marius Stelian Ilie

**Affiliations:** Department of Parasitology and Parasitic Disease, Faculty of Veterinary Medicine, University of Life Sciences “King Mihai I” from Timisoara, Timisoara, Romania

**Keywords:** domestic pig, Europe, nematoda, protozoa, zoonotic species

## Abstract

**Introduction:**

Gastrointestinal parasitic infections remain an important concern in European pig production due to their potential impact on animal health, productivity, and, in the case of zoonotic species, public health. This systematic review investigated the occurrence of gastrointestinal helminths and enteric protists in domestic pigs across Europe and analysed the factors associated with their prevalence.

**Methods:**

A total of 28 studies published between 2010 and 2026 were included, identified through a structured search strategy conducted in three scientific databases.

**Results:**

The included studies reported a wide diversity of helminths and enteric protists in pigs raised under intensive, extensive, organic, and alternative production systems. Among helminths, *Ascaris suum, Trichuris suis*, and *Oesophagostomum* spp. were the most frequently identified species, whereas *Balantioides coli, Cryptosporidium* spp., *Cystoisospora suis*, and *Blastocystis* sp. represented the main enteric protists reported.

**Discussion:**

Overall, gastrointestinal parasites and enteric protists remain widely distributed in domestic pigs across Europe, with prevalence influenced by production system, management practices, host age, and diagnostic methodology. The identification of species with zoonotic potential also underlines the importance of continuous epidemiological surveillance within the One Health framework.

## 1 Introduction

Pig (*Sus scrofa*) production plays a key role in the European livestock sector, with the European Union ranking among the world’s leading producers and exporters of pork (European Commission, ; Hinney et al., 2021; Renaudeau and Dourmad, 2022). Despite major advances in production efficiency and animal health over recent decades, gastrointestinal parasitic infections remain endemic in many pig populations and continue to affect productivity, animal welfare and farm profitability (Hinney et al., 2020; Stensvold et al., 2021; Cioacă et al., 2025; Han et al., 2025). In addition, several pig-associated parasites have zoonotic potential, making porcine parasitic infections relevant not only for veterinary medicine but also for public health (Stensvold et al., 2021; Ballweber, 2024a).

The epidemiology of gastrointestinal parasites in pigs is shaped by multiple interacting factors, including production system (intensive, outdoor, and organic), farm management and hygiene, environmental contamination, and the diagnostic methods applied, resulting in substantial heterogeneity in reported prevalence and species distribution across European countries (Stensvold et al., 2021; Ballweber, 2024a; Cioacă et al., 2025). Differences in diagnostic sensitivity, particularly between coprological, immunological and molecular approaches, further contribute to variation in detection and reporting, limiting the comparability of epidemiological data.

Gastrointestinal parasites of domestic pigs belong to diverse taxonomic groups, including helminths, coccidia and other intestinal protists, as well as microsporidia. Among helminths, nematodes such as *Ascaris suum*, *Trichuris suis*, and *Oesophagostomum* spp. are well recognised in pigs. *Ascaris suum* is an ascarid with a direct life cycle, transmitted through the ingestion of embryonated eggs from the contaminated environment. Hepato-pulmonary larval migration may cause hepatic and pulmonary lesions, while intestinal infections may adversely affect productive performance. Coprological diagnosis of patent infections is based primarily on the detection of eggs in faeces; however, differentiation between *A. suum* and *A. lumbricoides* cannot be reliably achieved on the basis of egg morphology alone. The close genetic relationship between the two species and evidence of cross-transmission between pigs and humans support the zoonotic relevance of *A. suum* (Dold and Holland, 2011; Leles et al., 2012; Vlaminck et al., 2014).

*Trichuris suis* is an intestinal nematode located predominantly in the caecum and colon and is transmitted through the ingestion of embryonated eggs. Infections with high parasite burdens may be associated with intestinal inflammation, diarrhoea, anaemia, and reduced growth (Beer, 1973; Andreasen et al., 2015; Bünger et al., 2022; Ballweber, 2024b). Coprological diagnosis is based on the detection of characteristic eggs with two polar plugs in faecal samples (Bünger et al., 2022; Ballweber, 2024b).

*Oesophagostomum* spp., particularly *O. dentatum* and *O. quadrispinulatum*, are intestinal strongyles of pigs, with adult worms located predominantly in the large intestine. Infection occurs through the ingestion of infective third-stage larvae, and larval development within the intestinal mucosa may induce inflammatory reactions and nodule formation. Strongylid-type eggs can be detected by coprological examination; however, species-level differentiation may require characterisation of larval stages or molecular methods (Gasser et al., 2007; Andreasen et al., 2015). The pig-adapted *Oesophagostomum* species are not generally regarded as important zoonotic agents, although their potential relevance to human infection remains incompletely characterised.

Porcine coccidia include *Cystoisospora suis* and *Eimeria* spp. *Cystoisospora suis* is the aetiological agent of neonatal porcine cystoisosporosis and primarily affects suckling piglets. Multiplication of the parasite within the epithelium of the small intestine may cause mucosal lesions, malabsorption, diarrhoea, and reduced growth performance. Coprological diagnosis is based on the detection of oocysts shed in faeces, while molecular methods enable specific identification of the parasite (Gasser et al., 2007; Shrestha et al., 2015). *Eimeria* spp. are also intestinal coccidia found in pigs, and oocysts represent the diagnostic stage shed in faeces (Han et al., 2025). Porcine coccidia are primarily important from an animal health perspective, and there is currently no convincing evidence supporting a zoonotic role for *Cys. suis*.

*Cryptosporidium* spp. are apicomplexan protists that infect the gastrointestinal tract of a wide range of vertebrates. In pigs, *Cry. suis* and *Cry. scrofarum* are frequently identified species, and their distribution may vary according to host age. Transmission occurs via the faecal–oral route through the ingestion of infective oocysts shed in faeces. Infections in pigs are often subclinical but may be associated with enteric manifestations, particularly in young animals. Diagnosis may include the detection of oocysts in faeces, whereas molecular methods enable the identification and differentiation of species and subtypes. Pigs may also harbour *Cry. parvum*, a species of recognised zoonotic relevance, while *Cry. suis* and *Cry. scrofarum* have been sporadically reported in humans. However, the role of pigs in zoonotic transmission should be interpreted on the basis of the available molecular and epidemiological evidence (Schubnell et al., 2016; Zahedi and Ryan, 2020).

Other intestinal protists reported in pigs include *Balantioides coli*, *Blastocystis* sp., *Giardia duodenalis*, and intestinal amoebae. *Balantioides coli* is an intestinal ciliate for which pigs are considered the principal reservoir hosts. Transmission occurs via the faecal–oral route through the ingestion of cysts, and both cysts and trophozoites may be identified in faecal samples. Infection in pigs is frequently asymptomatic, although the parasite may be associated with intestinal lesions and clinical manifestations under certain conditions. *Balantioides coli* has well-documented zoonotic relevance, with human infection associated with direct or indirect exposure to sources contaminated with porcine faecal material (Schuster and Ramirez-Avila, 2008; Ponce-Gordo and García-Rodríguez, 2021).

*Blastocystis* sp. is characterised by considerable genetic diversity, with several subtypes identified in pigs. Transmission is considered to occur predominantly via the faecal–oral route, and diagnosis can be performed using microscopic examination and molecular methods, with the latter enabling subtype differentiation. The clinical significance of *Blastocystis* colonisation remains incompletely understood, and some subtypes identified in pigs have also been reported in humans, supporting the need for further investigation of their zoonotic potential (Stensvold and Clark, 2016; Asghari et al., 2021).

*Giardia duodenalis* is a flagellated protist of the small intestine with a life cycle comprising trophozoites and cysts. Transmission occurs predominantly via the faecal–oral route through the ingestion of cysts, and cysts and, under certain conditions, trophozoites may be detected in faecal samples. Molecular methods enable the characterisation of assemblages, of which Assemblages A and B are of zoonotic relevance, whereas Assemblage E is frequently associated with animals, including pigs (Feng and Xiao, 2011; Asghari et al., 2023).

Intestinal amoebae identified in pigs include species of the genus *Entamoeba*, particularly *E. polecki*, as well as other taxa detected using morphological or molecular methods. Cysts may be identified in faeces; however, species differentiation based solely on morphology can be difficult, and molecular methods enable more precise taxonomic and genetic characterisation. For *E. polecki*, ST1 and ST3 have been identified in pigs and have also been reported in humans, indicating the relevance of investigating their zoonotic potential. However, the detection of the same subtypes in pigs and humans does not, by itself, demonstrate direct transmission between the two hosts (Hirashima et al., 2017; Stensvold et al., 2021; Wang et al., 2022).

*Enterocytozoon bieneusi* is an obligate intracellular microsporidian with a broad host range, including humans and numerous domestic and wild animals. Transmission occurs predominantly via the faecal–oral route through infective spores shed in faeces. In pigs, infection is frequently asymptomatic. Diagnosis and genetic characterisation rely primarily on molecular methods, with genotyping commonly performed through analysis of the internal transcribed spacer (ITS) region of ribosomal DNA. Pigs may harbour genotypes that have also been reported in humans, highlighting the potential zoonotic relevance of porcine *E. bieneusi* infections and the value of molecular characterisation for evaluating epidemiological relationships between hosts (Santín and Fayer, 2011; Li et al., 2020; Taghipour et al., 2022).

From a One Health perspective, pigs represent an important interface between animals, humans and the environment, as they may act as reservoirs for pathogens that contaminate soil, water and food chains, thereby facilitating human exposure (Gebreyes et al., 2014; Destoumieux-Garzón et al., 2018). The One Health framework explicitly recognises that diseases emerging at the human - animal - environment interface require integrated surveillance and coordinated control strategies rather than sector-specific approaches (Food and Agriculture Organization of the United Nations, ; World Health Organization, ).

Although numerous studies have investigated gastrointestinal parasites in pigs across Europe, the available evidence remains fragmented, with inconsistent methodologies, reporting standards and geographical coverage. To date, no comprehensive synthesis has systematically integrated these data to evaluate prevalence patterns, spatial distribution and diagnostic approaches within a unified, transparent framework.

Based on this framework, the aim of this study was to provide a descriptive epidemiological synthesis of the available data on gastrointestinal parasites in domestic pigs across Europe. The review aimed to address the following research question: “What are the prevalence, geographical distribution, and diagnostic approaches used for the detection of gastrointestinal parasites in domestic pigs (*Sus scrofa domesticus*) in Europe?”.

## 2 Materials and methods

### 2.1 Search strategy

In this context, a systematic review is necessary to synthesise the available evidence in a transparent, reproducible, and methodologically sound manner. Accordingly, the present systematic review was conducted and reported in accordance with the PRISMA 2020 guidelines (Preferred Reporting Items for Systematic Reviews and Meta-Analyses) (Page et al., 2021).

In accordance with the PICO framework, the population (P) consisted of domestic pigs (*Sus scrofa domesticus*) in Europe, the exposure (I/E) was gastrointestinal parasitic infection, the comparison (C) included differences between production systems and diagnostic approaches, and the outcome (O) was the reported prevalence and distribution of these parasites.

A comprehensive literature search was conducted to identify relevant studies in three international scientific databases: PubMed, Web of Science (WOS), and ScienceDirect.

The search covered articles published in English between January 2010 and January 2026.

Articles were identified using Medical Subject Headings (MeSH) terms and free-text keywords, applied either individually or in combination using Boolean operators (AND/OR/NOT). The search strategy included terms such as “pig”, “swine”, “porcine”, “gastrointestinal parasites”, “intestinal parasites”, “helminths”, “protozoa”, “coccidia”, “*Ascaris suum*”, “*Trichuris suis*”, “*Cryptosporidium*”, “*Giardia*”, “*Blastocystis*”, “*Balantioides coli*”, “*Strongyloides ransomi*”, “*Hyostrongylus rubidus*”, “*Globocephalus*” and “Europe”.

The main search strings included:

- (*pig* OR *swine* OR porcine) AND (parasit* OR helminth* OR protozoa* OR coccid*) AND (gastrointestinal OR intestinal OR enteric OR gut) AND (Europe OR European) NOT (trichinell* OR toxoplas* OR “wild boar” OR feral pig* OR feral swine OR boar*);
- (“*Swine*”(MeSH) OR *pig* OR *swine* OR *porcine)* AND *(“Intestinal Diseases, Parasitic*”(MeSH) OR “*Helminthiasis*”(MeSH) OR “*Nematode Infections*”(MeSH) OR “*Protozoan Infections*”(MeSH) OR parasite* OR “gastrointestinal parasite*” OR “intestinal parasite*” OR helminth* OR protozoa* OR coccid*) AND (“Europe”(MeSH) OR Europe OR European) NOT (trichinell* OR toxoplas* OR “wild boar” OR feral pig* OR feral swine OR boar*);
- (*pig* OR *swine* OR *porcine)* AND *(*“*Ascaris suum*” OR “*Trichuris suis*” OR *Oesophagostomum* OR “*Strongyloides ransomi*” OR “*Hyostrongylus rubidus*” OR *Globocephalus* OR *Eimeria* OR “*Cystoisospora suis*” OR “*Isospora suis*” OR *Cryptosporidium* OR *Giardia* OR *Blastocystis* OR “*Balantioides coli*” OR “*Balantidium coli*”) AND (*Europe* OR *European*) NOT (trichinell* OR toxoplas* OR “wild boar” OR feral pig* OR feral swine OR boar*).

As each database uses different indexing systems and imposes specific limitations regarding the number of searchable terms and Boolean combinations, the search syntax was adapted accordingly for each database.

*Trichinella* spp. and *Toxoplasma gondii* were excluded from the search strategy because the present review focused specifically on enteric gastrointestinal parasites of domestic pigs. Although both species are of major zoonotic importance, they primarily exhibit tissue tropism and extraintestinal pathogenesis and are therefore not considered true gastrointestinal parasites from an epidemiological and clinical perspective.

### 2.2 Inclusion and exclusion criteria

Studies were considered eligible for inclusion if they met all of the following criteria:

- reported data on gastrointestinal parasites in domestic pigs (*Sus scrofa domesticus*);
- were conducted in European countries;
- were published in English between January 2010 and January 2026;
- were original peer-reviewed research articles; and
- provided quantitative data regarding parasite occurrence, detection, or prevalence.

Geographical eligibility was determined according to the location in which each study was conducted, using the conventional geographical boundary between Europe and Asia as defined by the European Environment Agency (EEA) (European Environment Agency, 1995). For transcontinental countries spanning both Europe and Asia, only studies conducted within the European geographical part of the country were considered eligible, provided that all other predefined inclusion criteria were met.

Both cross-sectional and observational epidemiological studies were considered eligible. Studies using coprological, microscopic, serological, or molecular diagnostic methods for the detection of gastrointestinal parasites were included.

Studies were excluded if they:

- were review articles, meta-analyses, case reports, conference abstracts, letters to the editor, or unpublished studies;
- focused on non-European regions;
- investigated animal species other than domestic pigs;
- investigated non-gastrointestinal parasites, bacterial or viral diseases, or ectoparasites;
- consisted exclusively of experimental, pharmacological, genetic, or phylogenetic investigations without epidemiological relevance;
- lacked full-text availability; or
- were duplicate records.

### 2.3 Data extraction

Data from the included studies were manually extracted and entered into a Microsoft Excel spreadsheet for organisation and analysis. The extracted information included: article title, first author, year of publication, country of study, study period, sample size, pig category, detected parasite species, diagnostic methods used, and the reported prevalence or occurrence data. Any inconsistencies identified during data extraction were resolved through discussion and consensus between the reviewers.

### 2.4 Study selection

All records identified through the primary search strategy were imported into the Zotero reference management software for organisation and duplicate removal prior to screening.

Titles and abstracts of all retrieved studies were independently screened by two reviewers according to the predefined eligibility criteria. Full-text articles considered potentially relevant were subsequently assessed for final eligibility. Any discrepancies arising during the study selection process were resolved through discussion and consensus between the reviewers, with consultation of an additional reviewer when necessary.

The study selection process is illustrated in a PRISMA 2020 flow diagram adapted to the specific objectives and eligibility criteria of the present review, while maintaining compliance with PRISMA reporting standards (Page et al., 2021).

### 2.5 Protocol registration

The review protocol was retrospectively registered in the Open Science Framework (OSF) (OSF registration: https://osf.io/fq86p/). The study was based exclusively on previously published data and did not involve clinical or intervention studies.

### 2.6 Data synthesis

The aim of this review was to provide a descriptive epidemiological synthesis of the available data on gastrointestinal parasites in domestic pigs across Europe. Due to the substantial heterogeneity among the included studies regarding study design, sampling strategies, diagnostic methods, pig categories, and production systems, a quantitative meta-analysis was considered inappropriate.

### 2.7 Risk of bias (quality) assessment

The methodological quality of the included studies was assessed using the Joanna Briggs Institute (JBI) Critical Appraisal Checklist for cross-sectional studies. This checklist comprises nine items, each evaluated using four response options: “Yes”, “No”, “Unclear”, and “Not Applicable”. The number of “Yes” responses was used to calculate the overall quality score for each study. Based on the total score, studies were categorised as high quality (7–9 points), moderate quality (4–6 points), or low quality (0–3 points). Quality assessment was conducted independently by two reviewers, and any discrepancies were resolved through discussion and consensus.

An overview of the methodological framework applied in the systematic review, including the search strategy, eligibility criteria, study selection, data extraction, quality assessment, and data synthesis, is presented in **Figure 1**.

**Figure 1:**
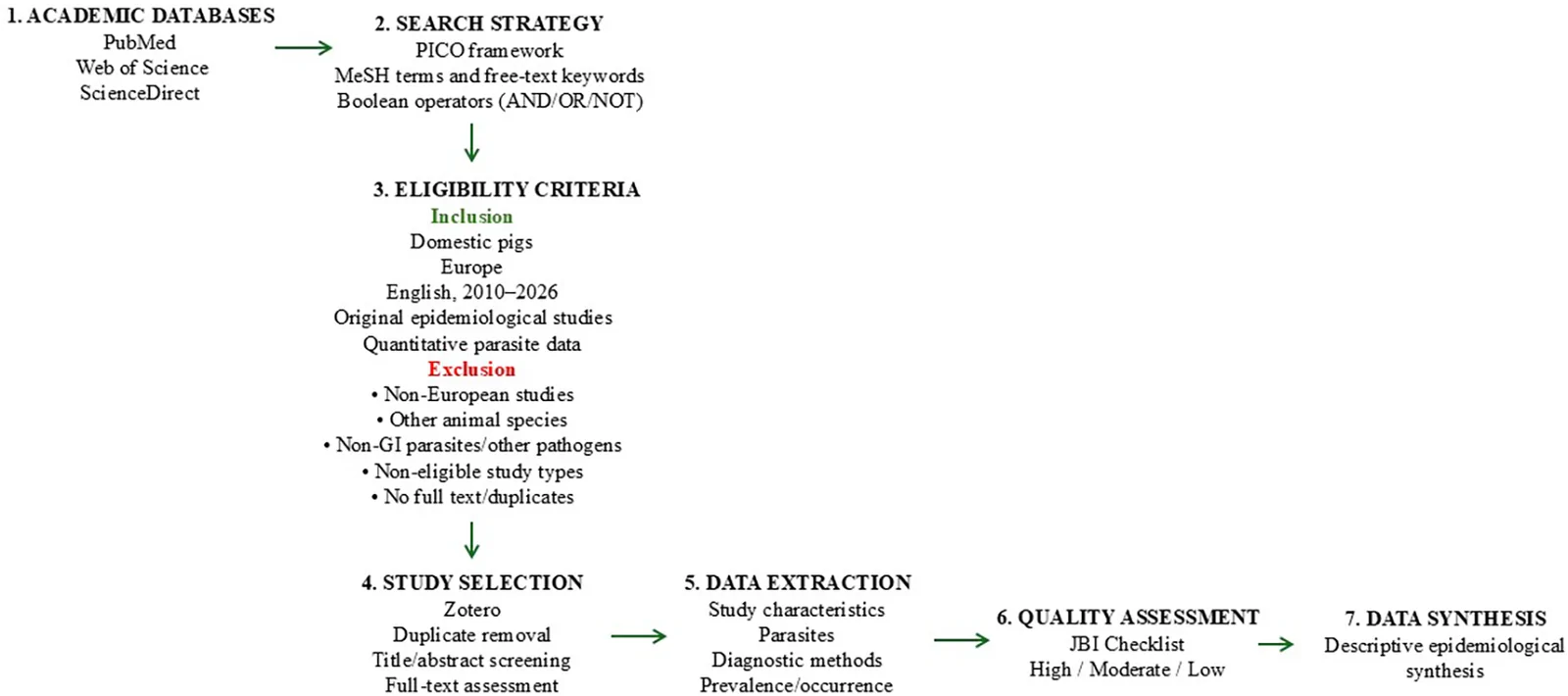
Schematic overview of the systematic review methodology and eligibility criteria.

## 3 Results

### 3.1 Search outcome

A total of 1,655 records were identified through database searching (PubMed = 522, Web of Science = 431, and ScienceDirect = 702). After the removal of duplicate records (n = 94), 1,561 records remained for title and abstract screening. Following the screening process, 1,518 records were excluded. The full texts of the remaining 43 articles were assessed for eligibility. Of these, 15 articles were excluded after full-text evaluation, and 28 studies met the inclusion criteria and were included in the final qualitative synthesis. The study selection process is summarised in **Figure 2**.

**Figure 2:**
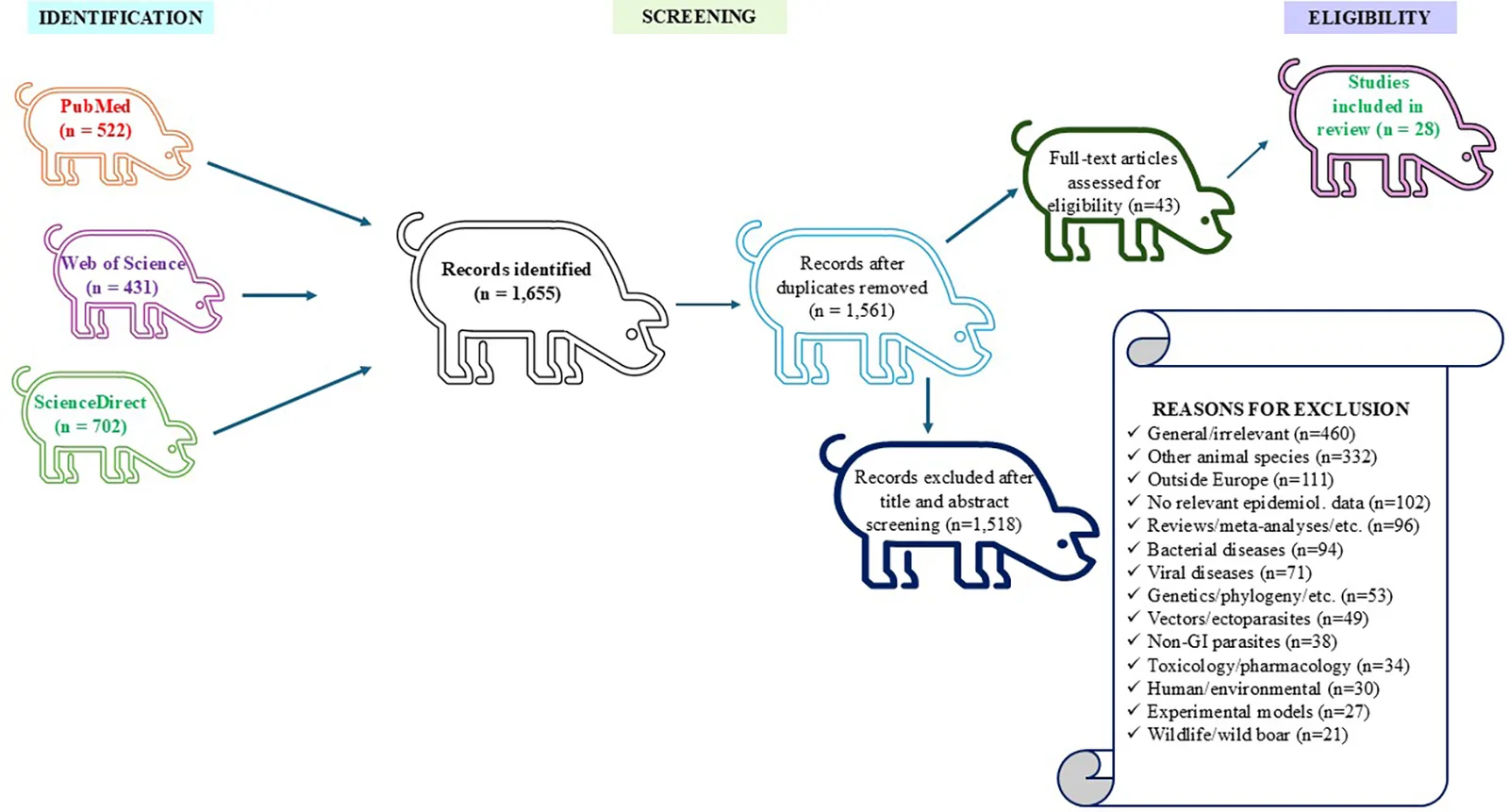
Adapted PRISMA 2020 flow diagram illustrating the identification, screening, eligibility assessment, and inclusion of studies in the present systematic review.

### 3.2 Characteristics of eligible studies

This systematic review included 28 studies conducted across 21 European countries. The included studies were published between 2010 and 2026. Sample sizes varied considerably between studies, ranging from 48 to 3,938 samples collected from pigs originating from various production systems, including intensive indoor systems, outdoor systems, free-range farms, organic farms, and mixed production systems.

With regard to geographical distribution, most studies were conducted in Spain (n = 3), Denmark (n = 3), Italy (n = 2), Poland (n = 2), Sweden (n = 2), Serbia (n = 2), Germany (n = 2), Romania (n=2), and the Czech Republic (n = 2), while other studies were conducted in Ukraine, France, Portugal, Greece, Ireland, United Kingdom, Norway, Switzerland, Slovakia, Austria, Belgium, and the Netherlands. Some multicountry studies were counted in more than one country category (**Figure 3**).

**Figure 3:**
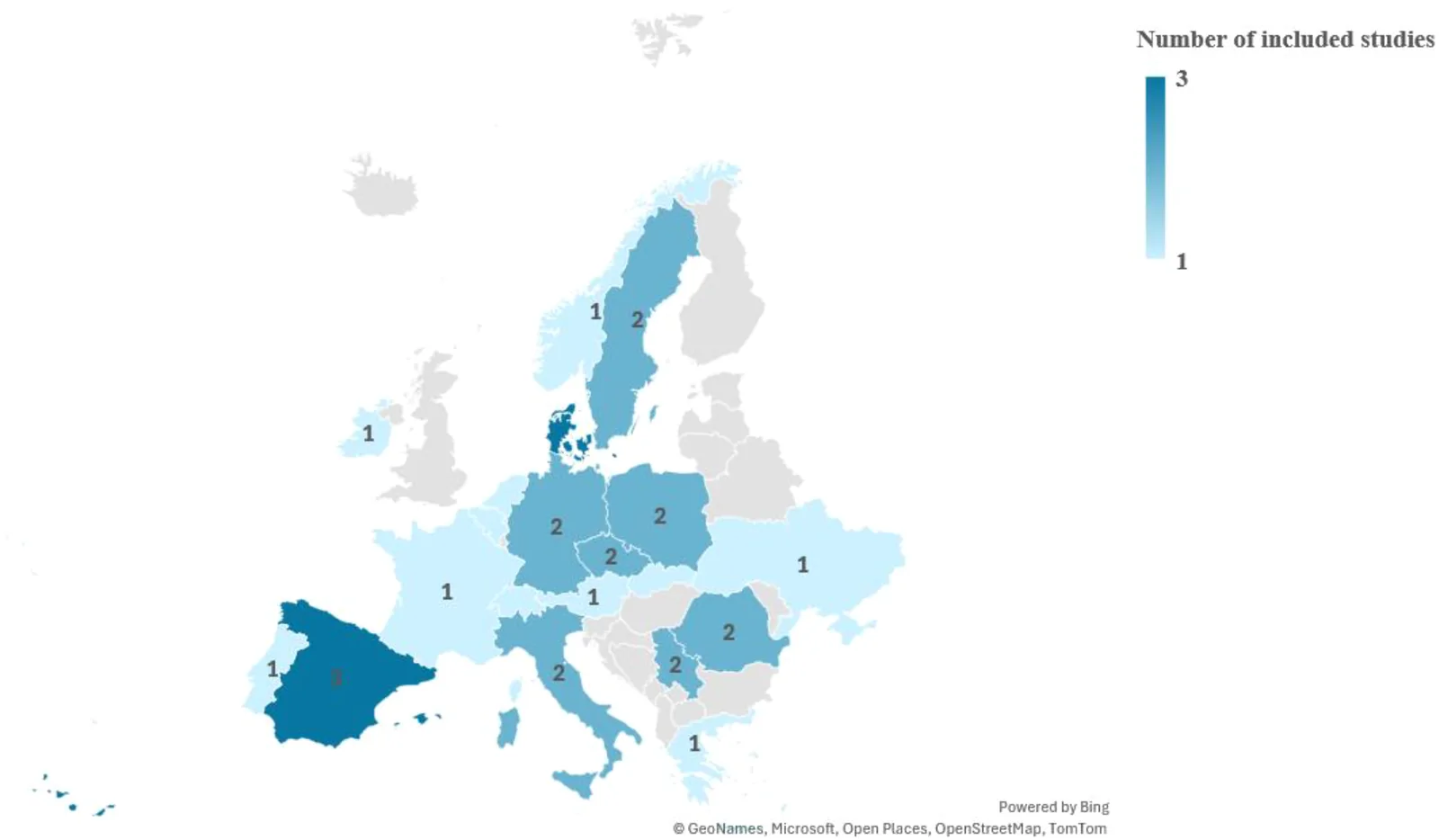
Geographical distribution of the studies included in the systematic review across Europe. Shading indicates the number of included studies reporting data from each country.

The included studies investigated a wide range of gastrointestinal parasites associated with domestic pigs, including *A. suum*, *Oesophagostomum* spp., *Cryptosporidium* spp., *Giardia* spp., *Blastocystis* sp., *B. coli*, *Cys. suis*, as well as other gastrointestinal helminths and protozoa. **Table 1** summarises the main characteristics and geographical distribution of the included studies.

**Table 1:** Characteristics and geographical distribution of studies investigating gastrointestinal parasites in domestic pigs.

Country	Period	Farming system	Diagnostic method	Parasite	Prevalence (%) [positive/total]	Genotype/Subtype (n)	Reference
Austria	2018	Conventional pig farms	Autofluorescence microscopy	*Cys. suis*	71.4% [5/7 farms]; 11.5% [9/78 litters]	–	(Hinney et al. (2020))
Belgium	–	Closed, multi-site farms	Autofluorescence microscopy	*Cys. suis*	72.7% [8/11 farms]; 43.6% [48/110 litters]		(Hinney et al. (2021))
Czech Republic	2018	Conventional pig farms	Autofluorescence microscopy	*Cys. suis*	70.6% [12/17 farms]; 32.9% [53/161 litters]		(Hinney et al. (2020))
	2009–2011	Commercial pig farms	Microscopy; nested PCR targeting 18S SSU rRNA; RFLP; species-specific PCR; *gp60* PCR; sequencing	*Cryptosporidium* spp.	Microscopy: 12.0% [194/1,620]; nested PCR: 21.8% [353/1,620]	–	(Němejc et al., 2013)
				*Cry. parvum*	0.06% [1/1,620]	IIaA16G1R1b [1/1]	
				*Cry. suis*	13.8% [224/1,620]	*-*	
				*Cry. scrofarum*	12.8% [208/1,620]		
				*Cry. suis+ Cry. scrofarum*	5.1% [82/1,620]		
				*Cry. muris*	0.2% [3/1,620]		
Denmark	–	Modern pig farms; loose-housed sows and gilts in large dynamic groups	Modified McMaster method (50 EPG)	*A. suum*	76% [60/79 farms]; 30% within infected farms	–	(Haugegaard (2010))
				*Oesophagostomum* spp.	15% [12/79 farms]; 50% within infected farms		
				*T. suis*	3/79 farms		
		Conventional pig farms (weaning units)	Metabarcoding targeting SSU rDNA	*B. coli*	57% [148/259]	Sequence type I: 64.2% of *B. coli*-positive samples; sequence type II: 35.8%	
				*Blastocystis* sp.	75% [193/259]	ST1: 28% [72/259]; ST3: 2% [6/259]; ST5: 68% [176/259]; ST15: 14% [36/259] Mixed subtypes: ST1+ST5 [62/193]; ST1+ST15 [29/193]; ST1+ST3 [1/193]; ST1+ST3+ST5 [1/193]; ST3+ST5+ST15 [1/193]	(Stensvold et al. (2021))
			Metabarcoding; real-time PCR	*Cryptosporidium* spp.	35% [90/259] by metabarcoding; 92% [239/259] by real-time PCR	–	
			Metabarcoding targeting SSU rDNA	*Cry. suis*	21% [54/259]		
				*Cry. scrofarum*	18% [46/259]		
				*E. hartmanni*	3% [8/259]		
				*E. polecki*	75% [193/259]	ST1: 40% [104/259]; ST3: 62% [161/259]; ST1+ST3: 37.4%	
			Real-time PCR; nested PCR; Sanger sequencing	*E. bieneusi*	92% [34/37]	EbpA	
			Real-time PCR	*G. duodenalis*	33% [86/259]	–	
			Metabarcoding	*I. bütschlii*	0.4% [1/259]	RL2 [1/259]	
	Sep 2011–Jun 2012	Organic pig farms	Immunofluorescence microscopy; PCR amplification; partial sequencing (SSU 18S rRNA and *hsp70*)	*Cryptosporidium* spp.	40.9% [350/856]	*-*	(Petersen et al. (2015))
				*Cry. scrofarum*	67.9% [38/56] among successfully characterised isolates		
				*Cry. suis*	32.1% [18/56] among successfully characterised isolates		
				*G. duodenalis*	14.0% [120/856]	Assemblage E: 84.6% [11/13]; Assemblage A: 15.4% [2/13]	
France	Jun 2020–Jun 2021	Alternative pig farms	McMaster method; ELISA	*A. suum*	Farm level: 16% [n=70 farms]; mean within-farm prevalence: sows 1.8%, growers 0.5%, finishers 2.9%; serology: 43% of finishers seropositive; 91% of farms with ≥1 seropositive finisher [n=65 farms]	–	(Delsart et al. (2022))
			McMaster method	*Eimeria* spp.	Farm level: 79% [n=70 farms]; mean within-farm prevalence: sows 29.1%, growers 14.2%, finishers 31.2%		
				*T. suis*	Farm level: 36% [n=70 farms]; mean within-farm prevalence: sows 2.6%, growers 5.1%, finishers 4.9%		
				*Oesophagostomum* spp./*Hyostrongylus rubidus*	Farm level: 47% [n=70 farms]; mean within-farm prevalence: sows 35.3%, growers 7.1%, finishers 10.0%		
				*Strongyloides* spp.	Detected in growers on 1 farm		
Germany	2018	Conventional pig farms	Autofluorescence microscopy	*Cys. suis*	42.9% [3/7 farms]; 15.5% [11/71 litters]	–	(Hinney et al., 2020)
	Feb 2022–Mar 2023	Alternative pig farms with outdoor areas	FLOTAC^®^	*A. suum*	27% [n=607]; farm level: 82% [14/17]; fatteners 37%		(Fischer et al. (2024))
				*T. suis*	5% [n=607]; farm level: 35% [6/17]		
			FLOTAC^®^; ITS-2 nemabiome metabarcoding; Illumina deep amplicon sequencing; phylogenetic analysis	Strongyles	32% [n=577]; farm level: 70% [12/17]; piglets: 28% [n not specified here]; fatteners: 28% [n not specified here]; adults: 44% [n not specified here]		
				*O. dentatum*	93.9% mean relative frequency		
				*O. quadrispinulatum*	5.9% mean relative frequency		
				*G. urosubulatus*	0.1% mean relative frequency		
Greece	Aug 2017–Jun 2018	Intensive farrow-to-finish farms	Flotation; Ziehl–Neelsen staining; microscopy	*A. suum*	3.7% [42/1150]	–	(Symeonidou et al. (2020))
				*B. coli*	37.8% [435/1150]		
				*Cys. suis*	6.0% [69/1150]		
				*Entamoeba* spp.	8.3% [95/1150]		
				*T. suis*	2.5% [29/1150]		
				*Oesophagostomum* spp.	1.4% [16/1150]		
Ireland	2023	Outdoor pig farm	McMaster flotation; morphological identification	*A. suum*	31% at both visits	–	(Senanayake et al. (2025))
				*Eimeria/Isospora* spp.	>80% at both visits		
				*T. suis*	9% at both visits		
				Strongyles	>80% at both visits		
Italy	Apr–Oct 2023	Intensive fattening pig farms; indoor housing	Sedimentation; FLOTAC^®^ (ZnSO_4_ and NaCl); conventional PCR targeting the ITS region, sequencing, cloning	*B. coli*	92.4% [813/880]; subset of 440 samples: sedimentation 95.9% [422/440], FLOTAC^®^-ZnSO_4_ 85.7% [377/440], FLOTAC^®^-NaCl 8.9% [39/440]	ITS type A [2/22]; type B [19/22]; types A+B [1/22]; variants A0, B0 and B1	(Allievi et al. (2025))
	Apr–Oct 2023	Intensive fattening farms	FLOTAC^®^ dual technique (NaCl and ZnSO_4_; sensitivity 2 EPG/OPG)	*A. suum*	7.6% [67/880]	–	(Allievi et al. (2024))
			FLOTAC^®^ dual technique (NaCl and ZnSO_4_; sensitivity 2 EPG/OPG)	*T. suis*	1.7% [15/880]		
			FLOTAC^®^ dual technique (NaCl and ZnSO_4_; sensitivity 2 EPG/OPG); sporulation and Sheather’s sugar flotation for coccidian identification	*Cys. suis*	0.9% [8/880]		
			FLOTAC^®^ dual technique; conventional PCR targeting the mitochondrial *nad1* gene and bidirectional sequencing	Hymenolepididae (*H. diminuta*)	1.8% [16/880]		
Netherlands	–	Predominantly piglet-producing farms	Autofluorescence microscopy	*Cys. suis*	25.8% [31/120 litters]; 50.0% [6/12 farms]	–	(Hinney et al. (2021))
Norway	Jun–Sep 2022; Nov 2023–Jan 2024	Intensive indoor pig production	Wet-mount microscopy; McMaster flotation; 18S rRNA PCR; Sanger sequencing	*B. coli*	PCR: 70.4% [88/125]; suckling piglets: 16.7% [2/12]; weaner piglets: 71.9% [23/32]; growers/finishers: 93.5% [29/31]; sows: 68.0% [34/50]. Wet-mount microscopy: 48.0% [12/25]; McMaster: 28.0% [7/25]	Type II	(Martinez-Contreras et al. (2026))
Poland	–	Pig farms of different sizes and production systems	McMaster method modified; morphological identification of eggs/oocysts; EPG/OPG determination	*A. suum*	Overall: 3.5% [n=1,119]; suckling piglets: 0.5% [n=367]; weaners: 1.2% [n=170]; fatteners: 6.5% [n=215]; sows: 3.8% [n=367]. Farm level: 28.6% [n=70]	–	(Kochanowski et al. (2017))
				Coccidia	Overall: 7.9% [n=1,119]; suckling piglets: 14.2% [n=367]; weaners: 3.5% [n=170]; fatteners: 3.7% [n=215]; sows: 6.0% [n=367]. Farm level: 42.9% [n=70]		
				*T. suis*	Overall: 2.1% [n=1,119]; suckling piglets: 0.5% [n=367]; weaners: 1.2% [n=170]; fatteners: 1.4% [n=215]; sows: 3.0% [n=367]. Farm level: 21.4% [n=70]		
				*Oesophagostomum* spp.	Overall: 24.2% [n=1,119]; suckling piglets: 13.1% [n=367]; weaners: 12.9% [n=170]; fatteners: 18.1% [n=215]; sows: 51.8% [n=367]. Farm level: 68.6% [n=70]		
				*Strongyloides* spp.	Overall: 2.0% [n=1,119]; suckling piglets: 3.3% [n=367]; weaners: 1.8% [n=170]; fatteners: 0% [0/215]; sows: 2.5% [n=367]. Farm level: 11.4% [n=70]		
	May–Sep 2008	Commercial pig farms	Nested PCR targeting 18S SSU rRNA; species-specific COWP-PCR; RFLP; sequencing of selected 18S SSU rRNA and COWP amplicons	*Cryptosporidium* spp.	Overall: 27.7% [46/166]; <1 month: 6.0% [2/33]; >1–2 months: 32.5% [14/43]; >2–3 months: 42.1% [16/38]; >3–5 months: 26.9% [14/52]; healthy pigs: 29.3% [34/116]; diarrhoeic pigs: 24.0% [12/50]; farm level: 53.8% [21/39]		(Rzeżutka et al. (2014))
			Nested PCR targeting 18S SSU rRNA; RFLP; sequencing	*Cry. suis*	4.2% [7/166]; farm level: 12.8% [5/39]		
				*Cry. scrofarum*	24.0% [40/166]; farm level: 51.2% [20/39]		
				*Cry. parvum*	0.6% [1/166]; farm level: 2.5% [1/39]		
Portugal	Oct 2020–Apr 2022	Industrial pig farms	Modified Ritchie concentration technique; autofluorescence microscopy	*Cys. suis*	85.2% [23/27]	–	(Nunes et al. (2023))
Romania	May 2020–May 2021	Extensive household rearing system	Flotation-Willis; direct; examination in Lugol’s solution; morphological identification	*A. suum*	12.9% [14/105]	–	(Cioacă et al. (2025))
				*B. coli*	35.5% [37/105]		
				*Eimeria*/*Isospora* spp.	9.7% [10/105]		
				*Hyostrongylus*/*Globocephalus* spp.	6.7% [7/105]		
				*Oesophagostomum* spp.	19.0% [20/105]		
				*T. suis*	9.7% [10/105]		
	2021 (all seasons)	Free-range farms	Centrifugal sedimentation; flotation-Willis; modified Ziehl–Neelsen staining; Blagg method; McMaster egg counting; faecal cultures; morphological identification	*A. suum*	Fatteners: 63.1% [202/320]; sows: 34.1% [109/320]		(Băieş et al. (2022))
				*B. coli*	Weaners: 70.3% [225/320]; fatteners: 72.5% [232/320]; sows: 62.1% [199/320]		
				*Cryptosporidium* spp.	Weaners: 18.1% [58/320]; sows: 9.3% [30/320]		
				*Eimeria* spp.	Weaners: 63.4% [203/320]; fatteners: 50.9% [163/320]; sows: 39.0% [125/320]		
				*Oesophagostomum* spp.	Weaners: 9.3% [30/320]; sows: 27.1% [87/320]		
				*T. suis*	Fatteners: 39.0% [125/320]		
				*S. ransomi*	Weaners: 3.7% [12/320]; sows: 1.5% [5/320]		
Serbia	Aug–Nov 2016	Commercial pig farms	Direct microscopy; xenic *in vitro* culture (XIVC); PCR targeting SSU rDNA; bilateral sequencing, phylogenetic analysis	*Blastocystis* sp.	Microscopy: 48/403 [11.9%]; XIVC: 79.2% [38/48]; PCR: 81.2% [39/48]	ST5 [11/11 sequenced pig isolates]	(Süli et al. (2021))
	2007–2011	Farms owned by individual breeders	Flotation - Willis; direct	*A. suum*	47.62% [491/1,031]	–	(Ilić et al. (2013))
Slovakia	–	Two pig farms; different hygiene standards	PCR; direct bidirectional sequencing of partial SSU rRNA gene; BLAST analysis and phylogenetic analysis	*Blastocystis* sp.	Overall: 12.0% [12/100]; Strážske farm: 28.6% [12/42] (piglets: 10.0% [2/20]; weaners: 25.0% [4/16]; sows: 100% [6/6]); Zemplínska Teplica farm: 0% [0/58]	ST5 [12/12]	(Danišová and Valenčáková, 2021)
Spain	2018	Conventional pig farms	Autofluorescence microscopy	*Cys. suis*	83.3% [15/18 farms]; 78.2% [229/293 litters]	–	(Hinney et al. (2020))
	Mar–Jun 2024	Commercial breeding farms	Formalin-ethyl acetate sedimentation method; immunofluorescence; nested PCR; Sanger sequencing	*Cryptosporidium* spp.	16.7% [12/72]; three-stage system: 21.9% [7/32]; two-stage system: 12.5% [5/40]; nursery: 25.0% [3/12]; fattening: 25.0% [3/12]; WTF: 17.4% [4/23]; lactation: 8.0% [2/25]; nested PCR: 66.7% [8/12 IF-positive]		(Garza-Moreno et al. (2025))
				*Cry. scrofarum*	87.5% [7/8] among successfully sequenced isolates		
				*Cry. suis*	12.5% [1/8] among successfully sequenced isolates		
	Oct–Nov 2015	Extensive Iberian pig farms	Nested PCR; bidirectional sequencing	*E. bieneusi*	22.6% [42/186]; sows: 31.6% [32/98]; fattening pigs: 11.4% [10/88]	EbpA [22/42]; O [8/42]; PigEb4 [3/42]; PigSpEb1 [3/42]; PigHN-II [2/42]; mixed EbpA + PigEb4 [4/42]; All Group 1	(Dashti et al. (2020))
Sweden	Oct 2017–Oct 2018	13 pig farms; 12 indoor farms and 1 organic farm with outdoor access; age-segregated production	Saline-glucose flotation; FITC-labelled monoclonal anti-*Cryptosporidium* antibodies; epifluorescence microscopy; nested PCR targeting 18S and 28S rRNA genes; sequencing	*Cryptosporidium* spp. (*Cry. suis, Cry. scrofarum, Cry. parvum*)	Overall: 25.2% [56/222]; piglets (0–5 weeks): 8.3% [4/48]; growers (6–12 weeks): 50.9% [29/57]; fatteners (13–24 weeks): 29.9% [20/67]; adults (>6 months): 6.0% [3/50]; herd level: 100% [13/13]		(Pettersson et al. (2020))
	2018-2020	Conventional indoor pig farms	Centrifugal flotation with saturated glucose–salt solution; modified McMaster technique (50 EPG)	*A. suum*	Post-weaning piglets: 0% [0/337]; growers: 1% [3/345]; fatteners: 9% [27/308]; dry sows: 8% [23/277]; pre-partum sows: 9% [32/348]		(Pettersson et al. (2021))
			Centrifugal flotation with saturated glucose–salt solution; modified McMaster technique (50 EPG); sporulation in 2% potassium dichromate	*Cys. suis*	Post-weaning piglets: 20% [67/337]; growers: 5% [16/345]; fatteners: 0.3% [1/308]; dry sows: 0% [0/277]; pre-partum sows: 0% [0/348]		
				*Eimeria* spp.	Post-weaning piglets: 2% [7/337]; growers: 3% [9/345]; fatteners: 2% [7/308]; dry sows: 18% [50/277]; pre-partum sows: 22% [76/348]		
			Centrifugal flotation with saturated glucose–salt solution; modified McMaster technique (50 EPG)	*T. suis*	Post-weaning piglets: 0% [0/337]; growers: 0% [0/345]; fatteners: 0% [0/308]; dry sows: 3% [7/277]; pre-partum sows: 2% [6/348]		
			Centrifugal flotation with saturated glucose–salt solution; modified McMaster technique (50 EPG); larval culture	*Oesophagostomum* spp.	Post-weaning piglets: 4% [12/337]; growers: 5% [18/345]; fatteners: 7% [20/308]; dry sows: 40% [111/277]; pre-partum sows: 41% [141/348]		
Switzerland	Jan–Dec 2014	Conventional pig farms	SAFC; microscopy	Amoebae	Overall: 25.6% [32/125]; suckling piglets: 7.7% [3/39]; weaned piglets: 26.7% [16/60]; fatteners: 50.0% [13/26]		(Schubnell et al. (2016))
			Combined sedimentation/flotation with ZnCl_2_; microscopy	*A. suum*	Overall: 0.8% [1/125]; fatteners: 3.8% [1/26]		
			SAFC; microscopy	*B. coli*	Overall: 29.6% [37/125]; suckling piglets: 5.1% [2/39]; weaned piglets: 36.7% [22/60]; fatteners: 50.0% [13/26]		
			Sedimentation/flotation with ZnCl_2_; sporulation in 2.5% potassium dichromate; microscopy	*Cys. suis*	Overall: 8.8% [11/125]; suckling piglets: 12.8% [5/39]; weaned piglets: 6.7% [4/60]; fatteners: 7.7% [2/26]		
			Modified Ziehl–Neelsen staining; microscopy; nested PCR targeting 18S rRNA; sequencing	*Cryptosporidium* spp. (*Cry. suis, Cry. scrofarum*)	Overall: 14.4% [18/125]; suckling piglets: 10.3% [4/39]; weaned piglets: 15.0% [9/60]; fatteners: 19.2% [5/26]		
			Sedimentation/flotation with ZnCl_2_; microscopy	*T. suis*	Overall: 1.6% [2/125]; suckling piglets: 2.6% [1/39]; weaned piglets: 1.7% [1/60]		
				Strongylida	Overall: 1.6% [2/125]; weaned piglets: 1.7% [1/60]; fatteners: 3.8% [1/26]		
United Kingdom	Jan–Dec 2007	Indoor- and outdoor-housed pigs	Fluorescent antibody test (FAT); PCR-RFLP; confirmatory sequencing at the 18S rRNA locus	*Cryptosporidium* spp.	38.6% [119/308]; sucklers: 20.0% [4/20]; weaners: 38.9% [14/36]; growers: 48.4% [45/93]; finishers: 37.0% [44/119]; adults: 30.8% [12/39]; farm level: 69.5% [50/72]		(Featherstone et al. (2010))
				*Cry. scrofarum*	64.1% [25/39]		
				*Cry. parvum*	20.5% [8/39]		
				*Cry. suis*	15.4% [6/39]		
Ukraine	Mar 2020–Mar 2022	Large (>100 sows), medium (25–100 sows), and small (<25 sows) pig farms	Direct microscopy	*B. coli*	Large: not detected. Medium: 0–2 mo: 5.7% [n=510]; 2–4 mo: 6.5% [n=630]; fattening: 5.2% [n=495]; sows: 2.1% [n=139]. Small: 0–2 mo: 11.2% [n=320]; 2–4 mo: 17.2% [n=295]; fattening: 12.9% [n=240]; sows: 9.5% [n=42]		(Bohach et al. (2023))
			Ethyl acetate–formalin concentration method; direct microscopy	*Blastocystis* sp.	Large: 0–2 mo: 1.9% [n=415]; 2–4 mo: 2.1% [n=380]; fattening: 1.7% [n=350]; sows: 4.1% [n=122]. Medium: 0–2 mo: 3.3% [n=510]; 2–4 mo: 4.1% [n=630]; fattening: 3.8% [n=495]; sows: 5.0% [n=139]. Small: 0–2 mo: 5.6% [n=320]; 2–4 mo: 7.1% [n=295]; fattening: 3.8% [n=240]; sows: 7.1% [n=42]		
			Native faecal smears; Kester and Romanovsky–Giemsa staining; microscopy	*Cryptosporidium* spp.	Large: 0–2 mo: 6.9% [n=415]; 2–4 mo: 3.1% [n=380]; fattening: 1.1% [n=350]; sows: 4.9% [n=122]. Medium: 0–2 mo: 13.1% [n=510]; 2–4 mo: 10.3% [n=630]; fattening: 0.4% [n=495]; sows: 3.6% [n=139]. Small: 0–2 mo: 12.8% [n=320]; 2–4 mo: 16.2% [n=295]; fattening: 2.5% [n=240]; sows: 4.7% [n=42]		
			McMaster method in Raynaud’s modification; microscopy	*Cys. suis*	Large: 0–2 mo: 29.7% [n=415]; 2–4 mo: 12.3% [n=380]; fattening: 1.7% [n=350]; sows: 5.7% [n=122]. Medium: 0–2 mo: 32.3% [n=510]; 2–4 mo: 14.4% [n=630]; fattening: 4.2% [n=495]; sows: 4.3% [n=139]. Small: 0–2 mo: 30.0% [n=320]; 2–4 mo: 20.6% [n=295]; fattening: 4.5% [n=240]; sows: 2.3% [n=42]		
				*Eimeria* spp.	Large: 0–2 mo: 23.2% [n=415]; 2–4 mo: 21.3% [n=380]; fattening: 8.0% [n=350]; sows: 8.1% [n=122]. Medium: 0–2 mo: 4.3% [n=510]; 2–4 mo: 29.4% [n=630]; fattening: 12.3% [n=495]; sows: 8.6% [n=139]. Small: 0–2 mo: 7.5% [n=320]; 2–4 mo: 13.5% [n=295]; fattening: 7.9% [n=240]; sows: 11.9% [n=42]		

### 3.3 Diagnostic methods

The studies included in this systematic review used a wide range of diagnostic methods for the detection and characterisation of gastrointestinal parasites and enteric protists in domestic pigs. Both conventional and molecular diagnostic approaches were reported across the included studies.

Conventional diagnostic techniques were the most frequently used methods and included light microscopy accompanied by concentration techniques (flotation, McMaster egg counting, coprological analysis, FLOTAC^®^), staining (Ziehl-Neelsen), and larval culture. These methods were primarily used for the detection and quantification of helminth eggs, protozoan oocysts, and cysts in faecal samples. Conventional methods were widely used due to their relatively low cost, ease of implementation, and suitability for large-scale epidemiological studies.

Molecular diagnostic methods included conventional polymerase chain reaction (PCR), real-time PCR, and metabarcoding, followed by Sanger sequencing or next-generation sequencing (NGS). These approaches enabled more accurate species-level identification and molecular characterisation of parasites such as *Cryptosporidium* spp., *Giardia* spp., *Blastocystis* sp., and *B. coli*. Molecular techniques also improved diagnostic sensitivity and allowed the detection of mixed or subclinical infections that may not be detectable using conventional microscopy alone.

Several studies combined molecular and conventional diagnostic methods in order to improve detection accuracy and provide a more comprehensive assessment of parasite diversity in pig production systems.

Among the included studies, 13 used only conventional methods, 4 used only molecular approaches, and 11 combined both diagnostic strategies.

### 3.4 Occurrence and diversity of gastrointestinal parasites in European pig populations

#### 3.4.1 Helminths

Gastrointestinal helminths were reported across a wide range of production systems, from conventional intensive farms to alternative systems, including outdoor and free-range production, as well as extensive household holdings. Among the taxa identified, *A. suum*, *T. suis*, and *Oesophagostomum* spp. were repeatedly reported across several of the investigated populations, whereas *S. ransomi*, *H. rubidus*, *Globocephalus* spp., and *Hymenolepis diminuta* were reported in a more limited number of studies and investigated populations (Haugegaard, 2010; Ilić et al., 2013; Kochanowski et al., 2017; Symeonidou et al., 2020; Pettersson et al., 2021; Băieş et al., 2022; Delsart et al., 2022; Allievi et al., 2024; Fischer et al., 2024; Cioacă et al., 2025). Considerable differences were observed between studies, both in prevalence and in the distribution of parasites across age categories and production systems.

For *A. suum*, high prevalence was reported in some populations raised in free-range systems or originating from individual pig keepers. On two free-range farms in Romania, the prevalence of *A. suum* was 63.1% [202/320] in fattening pigs and 34.1% [109/320] in sows, while *T. suis* was identified in 39.0% [125/320] of fattening pigs (Băieş et al., 2022). In Serbia, *A. suum* was detected in 47.62% [491/1,031] of pigs originating from individual pig keepers (Ilić et al., 2013). By contrast, in an extensive household production system investigated in Romania, prevalence was 12.9% [14/105] for *A. suum*, 19.0% [20/105] for *Oesophagostomum* spp., and 9.7% [10/105] for *T. suis*, while eggs attributed to *Hyostrongylus/Globocephalus* spp. were identified in 6.7% [7/105] of samples (Cioacă et al., 2025).

Higher prevalence of *Oesophagostomum* spp. among adult animals was observed in studies conducted in Sweden and Poland. On conventional indoor farms in Sweden, prevalence increased across age categories, from 4% [12/337] in post-weaning piglets, 5% [18/345] in growers, and 7% [20/308] in fattening pigs to 40% [111/277] in dry sows and 41% [141/348] in pre-partum sows (Pettersson et al., 2021). In the same population, *A. suum* was not detected in post-weaning piglets and remained at 1–9% in the other age categories, whereas *T. suis* was identified only in sows, with prevalence values of 2–3% (Pettersson et al., 2021). A similar pattern was observed in Poland, where the overall prevalence of *Oesophagostomum* spp. were 24.2% (n = 1,119) but reached 51.8% in sows (n = 367); at farm level, the parasite was detected on 68.6% of the 70 farms investigated (n = 70). The overall prevalence of *A. suum* and *T. suis* was 3.5% (n = 1,119) and 2.1% (n = 1,119), respectively, while *Strongyloides* spp. were identified in 2.0% (n = 1,119) of the animals (Kochanowski et al., 2017).

In alternative production systems with outdoor access, some of the included studies directly assessed the relationship between management practices and helminth infections. In the population investigated by Fischer et al. (2024), *A. suum*, strongyles, and *T. suis* were detected in 27% (n = 607), 32% (n = 577), and 5% (n = 607) of samples, respectively, while at farm level they were reported on 82% [14/17], 70% [12/17], and 35% [6/17] of farms, respectively (n = 17 farms). Pasture access was associated with higher odds of infection with *A. suum* and strongyles, whereas free-range and deep-litter systems were associated with higher odds of infection with strongyles and *T. suis* compared with shallow-litter systems. On intensive fattening farms, *A. suum* and *T. suis* were identified in 7.6% [67/880] and 1.7% [15/880] of the pigs examined, respectively, while larger farm size and the use of an all-in/all-out system were associated with a lower risk of nematode infection. Parasitic elements attributed to the family Hymenolepididae were also detected in 1.8% [16/880] of the animals, and molecular analysis confirmed the presence of *H. diminuta* (Allievi et al., 2024).

Helminth prevalence also varied considerably across other production systems and populations. On an outdoor farm, strongyles were detected in more than 80% of the animals evaluated at both sampling visits (n = 65; 37 at the first visit and 28 at the second visit), *A. suum* in 31%, and *T. suis* in 9% (Senanayake et al., 2025). On intensive farrow-to-finish farms, prevalence was 3.7% [42/1,150] for *A. suum*, 2.5% [29/1,150] for *T. suis*, and 1.4% [16/1,150] for *Oesophagostomum* spp (Symeonidou et al., 2020)., whereas on conventional farms, *A. suum* was identified in 0.8% [1/125], *T. suis* in 1.6% [2/125], and Strongylida in 1.6% [2/125] of the animals investigated (Schubnell et al., 2016). Differences between these findings should be interpreted with caution, as the studies varied in terms of the populations investigated, age structure, unit of analysis, sampling strategies, and diagnostic methods.

Associations between management characteristics and helminth infections were not consistent across the included studies. In an investigation conducted on modern sow farms, *A. suum* was identified on 76% [60/79] of farms, *Oesophagostomum* spp. on 15% [12/79], and *T. suis* on 3 of the 79 farms; farm-level prevalence was not associated with housing system, farm size, or the degree of contact with faecal material (Haugegaard, 2010). Together with the specific associations reported in other populations, these findings suggest that the relationship between management and helminth infections should be interpreted within the context of each study and cannot be generalised across all production systems.

Molecular methods further enabled characterisation of strongyle community composition beyond identification based on egg morphology. ITS-2 nemabiome metabarcoding identified *Oesophagostomum dentatum* as the major component, with a mean relative frequency of 93.9%, followed by *O. quadrispinulatum* (5.9%) and *Globocephalus urosubulatus* (0.1%). These values represent relative frequencies within the nemabiome rather than individual-level prevalence. The study also reported *G. urosubulatus* for the first time in domestic pigs in Europe (Fischer et al., 2024).

#### 3.4.2 Coccidia

Coccidia were reported across several production systems, including conventional and industrial farms as well as alternative, outdoor, free-range, and extensive systems. Among the taxa identified, *Cys. suis* and *Eimeria* spp. occurred in several of the populations investigated, with prevalence varying according to age category and the epidemiological unit analysed. For *Cys. suis*, investigations conducted at farm and litter level demonstrated the presence of the parasite in piglet populations across several European countries, while studies analysing age categories separately found higher prevalence in younger animals in certain populations (Shrestha et al., 2015; Hinney et al., 2020; Hinney et al., 2021; Pettersson et al., 2021).

Studies evaluating *Cys. suis* at farm and litter level showed considerable variation even when the same diagnostic method was used. On conventional farms, farm-level prevalence was 71.4% [5/7], 70.6% [12/17], 42.9% [3/7], and 83.3% [15/18] in Austria, the Czech Republic, Germany, and Spain, respectively, while litter-level prevalence ranged from 11.5% [9/78] to 78.2% [229/293] (Hinney et al., 2020). In a subsequent investigation, farm-level prevalence was 72.7% [8/11] on closed multi-site farms in Belgium and 50.0% [6/12] on predominantly piglet-producing farms in the Netherlands, with corresponding litter-level values of 43.6% [48/110] and 25.8% [31/120] (Hinney et al., 2021). A farm-level prevalence of 85.2% [23/27] was also recorded in industrial production systems (Nunes et al., 2023).

Prevalence estimates at the individual-animal level differed considerably from those reported at farm or litter level and are not directly comparable because they refer to different epidemiological units. *Cys. suis* was identified in 6.0% [69/1,150] of pigs on farrow-to-finish farms and in 0.9% [8/880] of pigs on intensive fattening farms (Symeonidou et al., 2020; Allievi et al., 2024). On conventional farms, an overall prevalence of 8.8% [11/125] was recorded, with values of 12.8% [5/39] in suckling piglets, 6.7% [4/60] in weaned piglets, and 7.7% [2/26] in fattening pigs (Schubnell et al., 2016).

Age represented another source of variation in *Cys. suis* prevalence in studies that analysed different animal categories separately. On conventional indoor farms, prevalence decreased from 20% [67/337] in post-weaning piglets to 5% [16/345] in growers and 0.3% [1/308] in fattening pigs, while the parasite was not detected in dry or pre-partum sows (Pettersson et al., 2021). Higher values in younger age categories were also observed across farms of different sizes. In piglets aged 0–2 months, prevalence was 29.7% (n = 415) on large farms, 32.3% (n = 510) on medium-sized farms, and 30.0% (n = 320) on small farms, with lower values in older age categories across all three farm-size groups (Bohach et al., 2023). Thus, in these two studies, the highest prevalence of *Cys. suis* occurred in younger pigs.

For *Eimeria* spp., the included studies did not demonstrate a consistent age-related distribution pattern. On two free-range farms, prevalence was 63.4% [203/320] in weaned pigs, 50.9% [163/320] in fattening pigs, and 39.0% [125/320] in sows (Băieş et al., 2022). On alternative farms, *Eimeria* spp. were identified on 79% of the 70 farms investigated, with mean within-farm prevalence of 29.1% in sows, 14.2% in growers, and 31.2% in fattening pigs (Delsart et al., 2022). On conventional indoor farms, prevalence was 2–3% in post-weaning piglets, growers, and fattening pigs, compared with 18% [50/277] in dry sows and 22% [76/348] in pre-partum sows (Pettersson et al., 2021).

Variation in *Eimeria* spp. according to both age and farm size was reported by Bohach et al. (2023). On large farms, prevalence was 23.2% (n = 415) in pigs aged 0–2 months and 21.3% (n = 380) in those aged 2–4 months, compared with 8.0% (n = 350) in fattening pigs and 8.1% (n = 122) in sows. On medium-sized farms, the highest value occurred in pigs aged 2–4 months (29.4%; n = 630), whereas on small farms prevalence ranged from 7.5% (n = 320) to 13.5% (n = 295) across the categories investigated.

Some investigations reported coccidia without identification to genus or species level, thereby limiting taxonomic comparisons. The category ‘Coccidia’ was reported in 7.9% of the 1,119 pigs examined and on 42.9% of the 70 farms investigated, with prevalence varying across age categories (Kochanowski et al., 2017). In other populations, the combined category *Eimeria*/*Isospora* spp. were identified in more than 80% of the animals examined at both sampling visits on an outdoor farm (Senanayake et al., 2025) and in 9.7% [10/105] of pigs in an extensive household production system (Cioacă et al., 2025). Because these studies reported the taxa as combined or unspecified categories, the findings cannot be retrospectively attributed to a particular genus or species.

Overall, coccidia were reported across diverse production systems, with no consistent prevalence pattern according to production system. *Cystoisospora suis* was detected at farm or litter level in industrial and conventional systems and on predominantly piglet-producing farms (Hinney et al., 2020; Hinney et al., 2021; Nunes et al., 2023), whereas high prevalence of *Eimeria* spp. or the combined category *Eimeria*/*Isospora* spp. occurred in some populations from alternative, free-range, and outdoor systems (Băieş et al., 2022; Delsart et al., 2022; Senanayake et al., 2025). Direct comparisons between studies should, however, be interpreted with caution, given differences in the animal categories examined, epidemiological units of analysis, farm structure, sampling strategies, and diagnostic methods.

#### 3.4.3 Other intestinal protists and microeukaryote

The intestinal protists identified in the included studies varied in both detection frequency and molecular characteristics. *Balantioides coli* and *Blastocystis* sp. were found in several pig populations and production systems, whereas *Entamoeba polecki*, *E. hartmanni*, and *Iodamoeba bütschlii* were characterised primarily through molecular investigations. Other studies reported amoeboid organisms at a generic level, without further taxonomic identification.

*Balantioides coli* *Balantioides coli* was identified in several pig populations, with prevalence varying across production systems and age categories. In conventional weaning units, SSU rDNA metabarcoding detected *B. coli* in 57% [148/259] of the pigs examined. Molecular characterisation revealed both sequence types, with type I detected in 64.2% of positive samples and type II in 35.8% (Stensvold et al., 2021). In free-range systems, prevalence was 70.3% [225/320] in weaned pigs, 72.5% [232/320] in fattening pigs, and 62.1% [199/320] in sows (Băieş et al., 2022). Prevalence values of 35.5% [37/105] and 37.8% [435/1,150] were recorded in an extensive household production system and on intensive farrow-to-finish farms, respectively (Symeonidou et al., 2020; Cioacă et al., 2025).

Age-related differences were observed in several populations. On conventional farms, the prevalence of *B. coli* increased from 5.1% [2/39] in suckling piglets to 36.7% [22/60] in weaned piglets and 50.0% [13/26] in fattening pigs, with an overall prevalence of 29.6% [37/125] (Schubnell et al., 2016). Differences according to farm size were also reported, with *B. coli* absent from large farms but present on medium-sized and small farms. On medium-sized farms, prevalence ranged from 2.1% in sows to 6.5% in pigs aged 2–4 months, while on small farms it ranged from 9.5% in sows to 17.2% in pigs aged 2–4 months (Bohach et al., 2023).

The diagnostic method represented another important source of variation in the detection frequency of *B. coli*. On intensive fattening farms, 92.4% [813/880] of samples were positive by sedimentation (Allievi et al., 2024). Among the subset of 440 samples analysed comparatively, 95.9% [422/440] were positive by sedimentation, 85.7% [377/440] by FLOTAC^®^ with ZnSO_4_, and 8.9% [39/440] by FLOTAC^®^ with NaCl. Of the 22 samples selected for molecular characterisation, ITS type B was identified in 19, type A in two, and types A and B concurrently in one sample; variants A0, B0, and B1 were also reported (Allievi et al., 2024). In a population reared under intensive indoor conditions, PCR detected *B. coli* in 70.4% [88/125] of the pigs examined. Prevalence ranged from 16.7% [2/12] in suckling piglets to 71.9% [23/32] in weaned piglets, 93.5% [29/31] in growers/fattening pigs, and 68.0% [34/50] in sows, while molecular characterisation identified type II (Martinez-Contreras et al., 2026).

*Blastocystis* sp. Molecular investigations of *Blastocystis* sp. revealed subtype diversity that was not reflected by overall prevalence alone. In conventional weaning units, SSU rDNA metabarcoding detected *Blastocystis* sp. in 75% [193/259] of the pigs examined. ST5 was identified in 68% [176/259] of the animals, ST1 in 28% [72/259], ST15 in 14% [36/259], and ST3 in 2% [6/259]. Subtype combinations were also observed, most frequently ST1+ST5 [62/193] and ST1+ST15 [29/193], together with ST1+ST3 [1/193], ST1+ST3+ST5 [1/193], and ST3+ST5+ST15 [1/193] (Stensvold et al., 2021).

ST5 was also identified in other pig populations. In one investigation, microscopic examination detected *Blastocystis* sp. in 48 of 403 samples (11.9%). Of these 48 microscopy-positive samples selected for further investigation, 38 (79.2%) were positive by xenic *in vitro* culture and 39 (81.2%) by PCR; all 11 sequenced porcine isolates were assigned to ST5 (Süli et al., 2021). Elsewhere, an overall prevalence of 12.0% [12/100] differed markedly between the two farms examined, with 28.6% [12/42] on one farm and no positive samples [0/58] on the other. On the positive farm, prevalence was 10.0% [2/20] in piglets, 25.0% [4/16] in weaned pigs, and 100% [6/6] in sows, and all 12 positive samples were assigned to ST5 (Danišová and Valenčáková, 2021). In an investigation without molecular characterisation, prevalence ranged from 1.7% to 7.1% according to age category and farm size (Bohach et al., 2023).

Intestinal amoebae. The level of taxonomic identification of intestinal amoebae differed considerably among studies. Metabarcoding identified *E. polecki* in 75% [193/259] of the pigs investigated, with ST1 detected in 40% [104/259] and ST3 in 62% [161/259], while the ST1+ST3 combination occurred in 37.4% of the animals. In the same population, *E. hartmanni* was identified in 3% [8/259] and *Iodamoeba bütschlii* in 0.4% [1/259]; ribosomal lineage RL2 was reported for the single sample positive for *I. bütschlii* (Stensvold et al., 2021).

Other investigations provided less specific taxonomic identification. Organisms belonging to the genus *Entamoeba* were detected in 8.3% [95/1,150] of the pigs examined without identification to species or subtype level; therefore, this prevalence cannot be attributed to a particular *Entamoeba* species (Symeonidou et al., 2020). The generic category ‘Amoebae’ was reported in 25.6% [32/125] of pigs in another population, with prevalence values of 7.7% [3/39] in suckling piglets, 26.7% [16/60] in weaned piglets, and 50.0% [13/26] in fattening pigs (Schubnell et al., 2016). Because these organisms were reported generically as amoebae, the findings cannot be retrospectively attributed to a particular genus or species.

Overall, findings for intestinal protists varied among the pig populations investigated and according to the diagnostic methods used. For *B. coli*, detection frequency differed considerably between sedimentation and the two FLOTAC^®^ methods (Allievi et al., 2025), while for *Blastocystis* sp., microscopy, xenic *in vitro* culture, and PCR yielded different detection frequencies within the same investigation (Süli et al., 2021). Molecular characterisation further documented the genetic diversity of several intestinal protists, including ITS types A and B for *B. coli*, ST1, ST3, ST5, and ST15 for *Blastocystis* sp., ST1 and ST3 for *E. polecki*, and ribosomal lineage RL2 for *I. bütschlii*.

#### 3.4.4 *Cryptosporidium* spp.

*Cryptosporidium* spp. were reported in pig populations from different production systems, with findings varying according to the population examined, age category, and diagnostic method used. The influence of the detection method was particularly evident in studies applying multiple techniques to the same population. On commercial farms in the Czech Republic, prevalence was 12.0% [194/1,620] by microscopy and 21.8% [353/1,620] by nested PCR (Němejc et al., 2013). A larger difference was recorded in conventional weaning units in Denmark, where *Cryptosporidium* spp. were detected in 35% [90/259] of pigs by metabarcoding and in 92% [239/259] by real-time PCR (Stensvold et al., 2021). These findings highlight the importance of considering the diagnostic method when comparing prevalence estimates across studies.

Age-related distribution was not uniform, although several investigations reported higher prevalence in intermediate growth stages than in very young piglets or adult animals. In Poland, prevalence increased from 6.0% [2/33] in piglets aged <1 month to 32.5% [14/43] in those aged >1–2 months and 42.1% [16/38] in those aged >2–3 months, before decreasing to 26.9% [14/52] in pigs aged >3–5 months (48 In Sweden, the highest prevalence was recorded in growers aged 6–12 weeks, at 50.9% [29/57], compared with 8.3% [4/48] in piglets aged 0–5 weeks and 6.0% [3/50] in adult animals (Pettersson et al., 2020). Growers also had the highest prevalence in the United Kingdom, at 48.4% [45/93], compared with 20.0% [4/20] in suckling piglets and 30.8% [12/39] in adults (Featherstone et al., 2010). Other populations showed different age-related distributions. In a conventional farming population, prevalence increased from 10.3% [4/39] in suckling piglets to 19.2% [5/26] in fattening pigs (Schubnell et al., 2016), while values of 18.1% [58/320] in weaned pigs and 9.3% [30/320] in sows were reported on two free-range farms (Băieş et al., 2022). Variation according to both age category and farm size was also documented, with the highest prevalence, 16.2%, observed in pigs aged 2–4 months on small farms (Bohach et al., 2023).

Molecular characterisation repeatedly identified *Cry. suis* and *Cry. scrofarum*, whereas *Cry. parvum* was reported in fewer investigations. In the Czech study, *Cry. suis* and *Cry. scrofarum* were identified in 13.8% [224/1,620] and 12.8% [208/1,620] of pigs, respectively, with co-infection involving both species detected in 5.1% [82/1,620]. *Cryptosporidium. muris* was identified in 0.2% [3/1,620] and *Cry. parvum* in 0.06% [1/1,620]; the single *Cry. parvum* isolate was assigned to 60 kDa glycoprotein (*gp60*) subtype IIaA16G1R1b (Němejc et al., 2013). *Cryptosporidium suis* and *Cry. scrofarum* were also identified in pig populations from Denmark, Switzerland, Sweden, and Spain, while *Cry. parvum* was additionally reported in Sweden and the United Kingdom (Featherstone et al., 2010; Petersen et al., 2015; Schubnell et al., 2016; Pettersson et al., 2020; Stensvold et al., 2021; Garza-Moreno et al., 2025).

In studies where only a subset of positive samples underwent molecular characterisation, species proportions should be distinguished from prevalence in the overall population. On organic farms in Denmark, the overall prevalence of *Cryptosporidium* spp. were 40.9% [350/856]; among the 56 molecularly characterised isolates, 67.9% [38/56] were assigned to *Cry. scrofarum* and 32.1% [18/56] to *Cry. suis* (Petersen et al., 2015). In the United Kingdom, 64.1% [25/39] of the 39 characterised isolates were assigned to *Cry. scrofarum*, 20.5% [8/39] to *Cry. parvum*, and 15.4% [6/39] to *Cry. suis* (Featherstone et al., 2010). In the Spanish study, seven [87.5%] of the eight successfully sequenced isolates were identified as *Cry. scrofarum* and one [12.5%] as *Cry. suis* (Garza-Moreno et al., 2025). These values describe the composition of the molecularly characterised isolates rather than the prevalence of individual species in the overall populations investigated.

With regard to production system, *Cryptosporidium* spp. were detected on conventional, commercial, organic, and free-range farms, with no consistent prevalence pattern associated with a particular system across the included studies. On commercial breeding farms, for instance, prevalence was 21.9% [7/32] in three-stage production systems and 12.5% [5/40] in two-stage systems, with variation also observed across production stages (Garza-Moreno et al., 2025). Overall, the included studies highlighted age-related variation, differences associated with diagnostic methods, and diversity in the *Cryptosporidium* species identified molecularly.

#### 3.4.5 Giardia duodenalis

*Giardia duodenalis* was investigated in two of the included studies, both conducted in pig populations in Denmark but involving different production systems and diagnostic methods. In conventional weaning units, *G. duodenalis* was detected by real-time PCR in 33% [86/259] of the pigs examined (Stensvold et al., 2021), whereas on organic farms the prevalence was 14.0% [120/856] (Petersen et al., 2015). In the latter study, molecular characterisation of a subset of 13 isolates identified Assemblage E in 84.6% [11/13] and Assemblage A in 15.4% [2/13]. These percentages describe the distribution of assemblages among the molecularly characterised isolates rather than the prevalence of Assemblages E and A among all 856 pigs examined. Overall, the available data on *G. duodenalis* were limited to two pig populations, with molecular characterisation documenting Assemblages E and A in the subset of isolates analysed.

#### 3.4.6 Enterocytozoon bieneusi

*Enterocytozoon bieneusi* was investigated in two of the included studies, which provided both prevalence data and information on genetic diversity. In conventional weaning units, *E. bieneusi* was detected by real-time PCR in 92% [34/37] of the 37 animals tested specifically for this parasite, while nested PCR followed by Sanger sequencing identified genotype EbpA (Stensvold et al., 2021). This prevalence refers to the subset of 37 animals tested for *E. bieneusi* rather than the entire population of 259 pigs included in the other analyses of the study.

In extensively raised Iberian pigs, *E. bieneusi* was detected in 22.6% [42/186] of the animals, with prevalence values of 31.6% [32/98] in sows and 11.4% [10/88] in fattening pigs (Dashti et al., 2020). Molecular characterisation of the 42 positive samples identified genotypes EbpA [22/42], O [8/42], PigEb4 [3/42], PigSpEb1 [3/42], and PigHN-II [2/42], together with four mixed infections involving EbpA and PigEb4 [4/42]; all genotypes belonged to Group 1. EbpA was the most frequently identified genotype, occurring alone in 22 of the 42 positive samples and in combination with PigEb4 in a further four.

### 3.5 Prevalence of major gastrointestinal parasites in Europe (comparative overview)

Overall, the included studies showed considerable heterogeneity in the prevalence of gastrointestinal parasites in domestic pigs across Europe. This variation was observed for both helminths and intestinal protists and should be interpreted in relation to differences in the populations investigated, age categories, production systems, epidemiological units of analysis, and diagnostic methods.

Among helminths, *A. suum*, *T. suis*, and *Oesophagostomum* spp. were repeatedly reported across the included studies, although prevalence varied considerably among populations and production systems. High prevalence was observed in some free-range and outdoor populations and on alternative farms, while high farm-level values were also reported in other production systems. In studies that statistically evaluated management factors, specific practices, including pasture access and housing or litter characteristics, were associated with the odds of infection with certain helminths (Fischer et al., 2024). Taken together, the available evidence does not support assigning a uniform level of risk to any particular production system.

Considerable variation was also evident among intestinal protists. *Balantioides coli* showed high prevalence in several populations from intensive, free-range, and conventional systems, while marked differences between diagnostic methods were documented in some investigations (Allievi et al., 2025).

For *Cryptosporidium* spp., several studies reported age-related variation, with higher prevalence in intermediate growth stages in certain populations, while comparisons across studies were further influenced by differences in diagnostic methods (Featherstone et al., 2010; Němejc et al., 2013; Rzeżutka et al., 2014; Pettersson et al., 2020; Stensvold et al., 2021). Regarding *Cys. suis*, high farm- and litter-level prevalence was reported in several piglet-producing populations, while studies examining age-related distribution found higher values in younger animals in certain populations.

Molecular characterisation revealed additional diversity beyond that conveyed by overall prevalence alone. For *Blastocystis* sp., ST1, ST3, ST5, and ST15 were identified, including combinations of subtypes, while ST5 was also documented in other pig populations (Danišová and Valenčáková, 2021; Süli et al., 2021; Stensvold et al., 2021). For *B. coli*, sequence types I and II, as well as ITS types A and B, were reported (Stensvold et al., 2021; Allievi et al., 2025; Martinez-Contreras et al., 2026).

Molecular characterisation of *Cryptosporidium* repeatedly identified *Cry. suis* and *Cry. scrofarum*, whereas *Cry. parvum* was reported in fewer investigations; *gp60* subtype IIaA16G1R1b was identified in a single *Cry. parvum* isolate (Němejc et al., 2013). Molecular diversity was also documented for *G. duodenalis*, with the identification of Assemblages E and A (Petersen et al., 2015), and for *E. bieneusi*, through the identification of multiple genotypes and mixed infections in the Iberian pig population investigated (Dashti et al., 2020).

Overall, no consistent prevalence pattern according to production system emerged across the included studies. Instead, the occurrence and prevalence of gastrointestinal parasites varied according to the populations and age categories investigated, management practices, epidemiological units of analysis, and diagnostic methods, while molecular investigations revealed additional genetic diversity within several taxa.

### 3.6 Risk of bias scores and overall methodological quality

A total of 28 studies were assessed for methodological quality. Of these, 15 studies were classified as high quality, while 12 were considered to be of moderate quality. No studies were categorised as low quality.

Overall, most studies demonstrated good methodological quality, particularly regarding the use of valid and reliable diagnostic methods (Q6 - Q7), which were consistently reported. Statistical analyses were also generally appropriate (Q8), especially in studies investigating epidemiological factors and parasite distribution.

However, several limitations were identified. The most common issue was related to the sampling methodology (Q2), which was frequently reported as unclear or insufficiently described, potentially affecting the representativeness of the study populations. In addition, the adequacy of the sample frame (Q1) varied across studies, with some investigations focusing on specific production systems, age categories, or geographical regions rather than broader pig populations. Information regarding response rates and the management of non-response (Q9) was generally lacking.

The detailed assessment for each JBI domain is presented in **Table 2** and **Supplementary Tables S1** and **S2**.

**Table 2:** Risk of bias scores and overall methodological quality of included studies.

Study	Score	Quality
(Allievi et al., 2025)	6	Moderate
(Allievi et al., 2024)	6	Moderate
(Băieş et al., 2022)	7	High
(Bohach et al., 2023)	6	Moderate
(Delsart et al., 2022)	8	High
(Fischer et al., 2024)	7	High
(Garza-Moreno et al., 2025)	5	Moderate
(Hinney et al., 2020)	7	High
(Ilić et al., 2013)	6	Moderate
(Kochanowski et al., 2017)	5	Moderate
(Nunes et al., 2023)	7	High
(Pettersson et al., 2020)	7	High
(Pettersson et al., 2021)	8	High
(Rzeżutka et al., 2014)	7	High
(Senanayake et al., 2025)	6	Moderate
(Stensvold et al., 2021)	7	High
(Symeonidou et al., 2020)	8	High
(Danišová and Valenčáková, 2021)	5	Moderate
(Dashti et al., 2020)	8	High
(Featherstone et al., 2010)	5	Moderate
(Hinney et al., 2021)	8	High
(Martinez-Contreras et al., 2026)	7	High
(Němejc et al., 2013)	8	High
(Petersen et al., 2015)	7	High
(Schubnell et al., 2016)	5	Moderate
(Süli et al., 2021)	6	Moderate
(Haugegaard, 2010)	4	Moderate
(Cioacă et al., 2025)	5	Moderate

## 4 Discussion

The present systematic review highlights the broad distribution and diversity of gastrointestinal parasites identified in domestic pigs across Europe. Helminths and intestinal protists were documented across a range of production systems, but their prevalence varied substantially among pig populations. Such variation needs to be considered in the context of animal age, husbandry and management practices, sampling strategies, epidemiological units of analysis, and diagnostic approaches. Differences between studies therefore cannot be attributed solely to geographical location or production system.

Among helminths, *A. suum*, *T. suis*, and *Oesophagostomum* spp. were repeatedly documented in conventional, intensive, alternative, outdoor, and free-range systems. The high prevalence observed in some populations with outdoor access is consistent with the importance of transmission through persistent parasitic stages in the environment; however, descriptive comparisons among farms do not in themselves demonstrate an effect of production system. Stronger evidence for the influence of management is provided by studies that statistically evaluated potential risk factors. On the alternative farms investigated by Fischer et al. (2024), pasture access was associated with higher odds of infection with *A. suum* and strongyles, while deep-litter and free-range systems were associated with higher odds of infection with strongyles and *T. suis* compared with shallow-litter systems. Cleaning frequency was also associated with infection with *A. suum* and strongyles. Larger farm size and the use of an all-in/all-out system, in turn, were associated with a lower risk of nematode infection in the population examined by Allievi et al. (2024). Collectively, these observations suggest that exposure to contaminated environments and specific management practices may contribute to variation in helminth infections, although the effects observed in individual studies should not be extrapolated to all outdoor, alternative, or intensive systems.

Molecular characterisation of strongyles provided information that could not be obtained from conventional identification based on egg morphology alone. Nemabiome analysis by Fischer et al. (2024) showed that *O. dentatum* accounted for 93.9% of the mean relative frequency of the sequences identified, compared with 5.9% for *O. quadrispinulatum* and 0.1% for *G. urosubulatus*. Importantly, these percentages describe the relative composition of the nematode community rather than individual-level prevalence. The identification of *G. urosubulatus*, reported as the first record of this species in domestic pigs in Europe, further illustrates the contribution of molecular approaches to resolving parasite diversity that may remain undetected when identification relies solely on egg morphology.

Intestinal protists were likewise widely distributed, but their detection varied substantially among pig populations. *Balantioides coli* occurred in intensive and conventional systems as well as in free-range populations, with high prevalence documented in some settings. Production system alone, however, does not adequately explain this variation. Allievi et al. (2025) found no significant association between the management variables evaluated and the presence of *B. coli*. At the same time, pronounced differences were recorded among diagnostic techniques, indicating that apparent prevalence may be strongly influenced by the method applied. Genetic analyses further revealed sequence types I and II and ITS types A and B across the populations examined (Stensvold et al., 2021; Allievi et al., 2024; Martinez-Contreras et al., 2026). These observations emphasise the need to interpret the epidemiology of *B. coli* by considering prevalence estimates together with diagnostic methodology and molecular data.

The combined influence of host age and diagnostic methodology was particularly evident for *Cryptosporidium* spp. Several studies found differences among age categories, with higher prevalence in intermediate growth stages in certain populations, although this distribution was not consistent across all investigations. Method-dependent differences were also substantial. In the Czech Republic, prevalence was 12.0% by microscopy and 21.8% by nested PCR (Němejc et al., 2013), while Stensvold et al. (2021) detected *Cryptosporidium* spp. in 35% of pigs by metabarcoding and 92% by real-time PCR. Reported prevalence should therefore not be interpreted exclusively as reflecting epidemiological differences among populations, since the performance and targets of the diagnostic techniques may substantially affect detection frequency. At species level, *Cry. suis* and *Cry. scrofarum* were repeatedly identified, whereas *Cry. parvum* occurred in fewer investigations. The identification of *gp60* subtype IIaA16G1R1b in a single *Cry. parvum* isolate (Němejc et al., 2013) further demonstrates the additional epidemiological information provided by molecular characterisation.

For *Cys. suis*, the available evidence confirms its occurrence in piglet populations, although the magnitude of prevalence differed substantially among studies. High farm- or litter-level prevalence was documented in some populations, and investigations separating animals by age generally found higher detection frequencies in younger pigs in certain populations. Interpretation across studies remains limited, however, because prevalence was variously expressed at farm, litter, or individual-animal level and because the populations and sampling strategies differed. High prevalence in individual investigations should consequently not be interpreted as evidence that *Cys. suis* occurs at uniformly high levels throughout European pig production.

Genetic diversity was also an important feature of several other intestinal protists. For *Blastocystis* sp., ST5 occurred in several pig populations, including all sequenced isolates in the studies from Serbia and Slovakia (Danišová and Valenčáková, 2021; Süli et al., 2021). The Danish population displayed broader diversity, with ST1, ST3, ST5, and ST15 and several subtype combinations detected within the same population (Stensvold et al., 2021). Characterisation of *G. duodenalis* revealed both Assemblage E, frequently associated with animals, and Assemblage A in a subset of isolates (Petersen et al., 2015). For *E. bieneusi*, genotype EbpA occurred in both populations subjected to molecular characterisation, while the Iberian pig population additionally harboured genotypes O, PigEb4, PigSpEb1, and PigHN-II, together with mixed EbpA+PigEb4 infections; all identified genotypes belonged to Group 1 (Dashti et al., 2020). Collectively, these findings show that prevalence estimates alone cannot capture the full epidemiological diversity of intestinal protists and underline the value of incorporating molecular characterisation into surveillance studies.

The detection in pigs of species, assemblages, subtypes, or genotypes that have also been reported in humans raises questions regarding the zoonotic relevance of some of the parasites identified. Examples among the included studies include *Cry. parvum*, Assemblage A of *G. duodenalis*, and certain subtypes or genotypes of *Blastocystis* sp. and *E. bieneusi*. Nevertheless, the occurrence of the same taxon or genetic variant in pigs and humans does not by itself demonstrate direct zoonotic transmission. Establishing shared transmission cycles requires comparative molecular investigations across host species supported by appropriate epidemiological data. The results of this review therefore support continued molecular surveillance, while interpretation of the zoonotic potential of these parasites should remain cautious.

Methodological variability represents a major limitation when comparing the included studies. Differences involved study design, sample size, age categories, production systems, sampling strategies, epidemiological units, and diagnostic techniques. The findings for *Cryptosporidium* obtained using microscopy, metabarcoding, and PCR provide a clear example, while a pronounced method-dependent effect was also evident for *B. coli*. Among the subset of 440 samples examined by multiple techniques in Allievi et al. (2025), sedimentation detected *B. coli* in 95.9%, compared with 85.7% using FLOTAC^®^ with ZnSO_4_ and 8.9% using FLOTAC^®^ with NaCl. Comparisons of prevalence estimates generated using different diagnostic approaches may therefore be misleading unless differences in diagnostic performance are considered.

The geographical coverage of the available evidence was also uneven. Although the included studies represented numerous European regions, both the number and characteristics of investigations differed among countries, with limited evidence available for some areas. This imbalance restricts the extent to which the findings can be considered representative of the overall European pig population. It also means that the absence or low reported frequency of a parasite in a particular region should not be interpreted as evidence of a genuinely restricted geographical distribution. Further epidemiological investigations based on well-defined sampling strategies and comparable diagnostic approaches would improve the characterisation of gastrointestinal parasite distribution in domestic pigs across Europe.

Overall, the findings support an integrated approach to parasitological surveillance in pig production. Surveillance and control strategies should account for the parasite involved, animal age, production system, and farm-specific conditions, as no single management factor emerged as an explanation for the distribution of all gastrointestinal parasites. The substantial methodological heterogeneity identified across studies also highlights the value of well-defined sampling strategies and comparable diagnostic approaches in future epidemiological investigations. Alongside conventional parasitological diagnostics, molecular methods can provide greater taxonomic resolution by identifying species, genotypes, subtypes, and mixed infections, thereby improving understanding of parasite diversity and transmission dynamics in pig populations.

### 4.1 Study limitations

This systematic review has several limitations that should be acknowledged when interpreting the findings. First, substantial methodological heterogeneity was observed among the included studies, particularly regarding study design, sampling strategies, pig production categories, and diagnostic techniques used for parasite detection. These differences may contribute to the wide variation in reported prevalence values and limit direct comparisons between studies.

Second, potential publication bias cannot be excluded, as studies reporting positive findings or higher parasite prevalence may be more likely to be published than studies reporting negative or low-prevalence results.

Third, the review was based on searches conducted in three major scientific databases, and relevant studies indexed in other databases or published in grey literature may not have been captured.

Finally, although a structured search strategy was applied, the selection of specific keywords and database indexing systems may have influenced the identification of eligible studies. These factors should be considered when interpreting the overall synthesis of the available evidence.

## 5 Conclusions

This systematic review provides an overview of gastrointestinal parasites reported in domestic pigs across Europe and highlights substantial variation in prevalence among the included studies. Differences were observed in relation to the populations investigated, host age, production and management conditions, and diagnostic approaches. Methodological differences, including those in sampling strategies, epidemiological units of analysis, and diagnostic methods, should therefore be considered when comparing prevalence estimates across European pig populations.

Several of the parasites identified are of recognised zoonotic relevance, while molecular characterisation also revealed assemblages, subtypes, and genotypes that have been reported in humans. However, their detection in pigs does not, by itself, demonstrate direct zoonotic transmission or establish shared transmission pathways between pigs and humans. Comparative molecular and epidemiological investigations across host populations are therefore needed to clarify the role of pigs in the transmission of these parasites.

Overall, the findings support continued parasitological surveillance based on well-defined sampling strategies and appropriate diagnostic methods, complemented by molecular characterisation where relevant. A One Health approach integrating animal, human, and environmental data may contribute to a more comprehensive understanding of parasite diversity, epidemiology, and potential transmission pathways at the human–animal–environment interface.

## Data Availability

The original contributions presented in the study are included in the article/**Supplementary Material**. Further inquiries can be directed to the corresponding author.
