# Supplementary material for: A systematic review of gastrointestinal parasites in Sus scrofa domesticus from Europe (2010 – 2026)
Source: Front Parasitol. 2026 Oct 1;5:1950643. doi: 10.3389/fpara.2026.1950643 (PMC13669084; doi:10.3389/fpara.2026.1950643)
Supplement: Supplementary file 1 [file SupplementaryFile1.pdf]

Supplementary Material

1 Supplementary Tables

Supplementary Table S1. JBI Critical Appraisal Criteria Used for Risk of Bias Assessment.

Question	Assessment criterion
**Q1**	Was the sample frame appropriate to address the target population?
**Q2**	Were study participants sampled in an appropriate way?
**Q3**	Was the sample size adequate?
**Q4**	Were the study subjects and the setting described in detail?
**Q5**	Was the data analysis conducted with sufficient coverage of the identified sample?
**Q6**	Were valid methods used for the identification of the condition?
**Q7**	Was the condition measured in a standard, reliable way for all participants?
**Q8**	Was there appropriate statistical analysis?
**Q9**	Was the response rate adequate, and if not, was the low response rate managed appropriately?

Supplementary Table S2. Detailed Risk of Bias Assessment of Included Studies.

Study	Q1	Q2	Q3	Q4	Q5	Q6	Q7	Q8	Q9
**(44)**	Unclear	Unclear	Yes	Yes	Yes	Yes	Yes	Yes	Unclear
**(45)**	Unclear	Unclear	Yes	Yes	Yes	Yes	Yes	Yes	Unclear
**(50)**	No	Yes	Yes	Yes	Yes	Yes	Yes	Yes	Unclear
**(59)**	Yes	Unclear	Yes	Yes	Yes	Yes	Yes	Unclear	Unclear
**(50)**	Yes	Yes	Yes	Yes	Yes	Yes	Yes	Yes	Unclear
**(41)**	Yes	Unclear	Yes	Yes	Yes	Yes	Yes	Yes	Unclear
**(54)**	No	Unclear	No	Yes	Yes	Yes	Yes	Yes	Unclear
**(5)**	Yes	Unclear	Yes	Yes	Yes	Yes	Yes	Yes	Unclear
**(52)**	Yes	Unclear	Yes	Yes	Yes	Yes	Yes	Unclear	Unclear
**(47)**	No	Unclear	Yes	Yes	Yes	Yes	Yes	Unclear	Unclear
**(49)**	Yes	Unclear	Yes	Yes	Yes	Yes	Yes	Yes	Unclear
**(56)**	Yes	Unclear	Yes	Yes	Yes	Yes	Yes	Yes	Unclear

**(57)**	Yes	Yes	Yes	Yes	Yes	Yes	Yes	Yes	Unclear
**(48)**	Yes	Unclear	Yes	Yes	Yes	Yes	Yes	Yes	Unclear
**(43)**	Yes	Unclear	No	Yes	Yes	Yes	Yes	Yes	Unclear
**(7)**	Yes	Unclear	Yes	Yes	Yes	Yes	Yes	Yes	Unclear
**(42)**	Yes	Yes	Yes	Yes	Yes	Yes	Yes	Yes	Unclear
**(53)**	No	No	Yes	Yes	No	Yes	Yes	Yes	Unclear
**(55)**	Yes	Yes	Yes	Yes	Yes	Yes	Yes	Yes	Unclear
**(58)**	Unclear	Unclear	Yes	Yes	Yes	Yes	Yes	No	Unclear
**(3)**	Yes	Yes	Yes	Yes	Yes	Yes	Yes	Yes	Unclear
**(46)**	Yes	Unclear	Yes	Yes	Yes	Yes	Yes	Yes	Unclear
**(37)**	Yes	Yes	Yes	Yes	Yes	Yes	Yes	Yes	Unclear
**(39)**	Yes	Yes	Unclear	Yes	Yes	Yes	Yes	Yes	Unclear
**(18)**	No	No	Yes	Yes	No	Yes	Yes	Yes	Unclear
**(51)**	Yes	Unclear	Unclear	Yes	Yes	Yes	Yes	Yes	Unclear
**(38)**	No	No	No	Yes	No	Yes	Yes	Yes	Unclear
**(6)**	Yes	Unclear	Unclear	Yes	Yes	Yes	Yes	Yes	Unclear

Note: Q1-Q9 correspond to the JBI critical appraisal criteria presented in Supplementary Table S1.
